# Pediatric abusive head trauma: visual outcomes, evoked potentials, diffusion tensor imaging, and relationships to retinal hemorrhages

**DOI:** 10.1007/s10633-023-09927-w

**Published:** 2023-03-07

**Authors:** John P. Kelly, Kenneth W. Feldman, Jason N. Wright, James B. Metz, Avery Weiss

**Affiliations:** 1 Roger H. Johnson Vision Clinic, Division of Ophthalmology, Seattle Children’s Hospital, Seattle, WA 98105 USA; 2 Department of Ophthalmology, University of Washington, Seattle, WA USA; 3 Safe Child and Adolescent Network, Seattle Children’s Hospital, Seattle, WA USA; 4 Division of General Pediatrics, University of Washington, Seattle, WA USA; 5 Division of Radiology, Seattle Children’s Hospital, Seattle, WA USA; 6 Department of Pediatrics, University of Vermont, Burlington, VT USA

**Keywords:** Abusive head trauma, Non-accidental trauma, Visual evoked potentials, Retinal hemorrhages, Diffusion tensor imaging, Pediatric ophthalmology

## Abstract

**Purpose:**

Function and anatomy of the visual system were evaluated in children with abusive head trauma (AHT). The relationships between retinal hemorrhages at presentation were examined with outcome measures.

**Methods:**

Retrospective review of data in children with AHT for 1) visual acuity at last follow-up, 2) visual evoked potentials (VEP) after recovery, 3) diffusion metrics of white matter tracts and grey matter within the occipital lobe on diffusion tensor imaging (DTI), and 4) patterns of retinal hemorrhages at presentation. Visual acuity was converted into logarithm of minimum angle of resolution (logMAR) after correction for age. VEPs were also scored by objective signal-to-noise ratio (SNR).

**Results:**

Of 202 AHT victims reviewed, 45 met inclusion criteria. Median logMAR was reduced to 0.8 (approximately 20/125 Snellen equivalent), with 27% having no measurable vision. Thirty-two percent of subjects had no detectable VEP signal. VEPs were significantly reduced in subjects initially presenting with traumatic retinoschisis or hemorrhages involving the macula (*p* < 0.01). DTI tract volumes were decreased in AHT subjects compared to controls (*p* < 0.001). DTI metrics were most affected in AHT victims showing macular abnormalities on follow-up ocular examination. However, DTI metrics were not correlated with visual acuity or VEPS. There was large inter-subject variability within each grouping.

**Discussion:**

Mechanisms causing traumatic retinoschisis, or traumatic abnormalities of the macula, are associated with significant long-term visual pathway dysfunction. AHT associated abnormalities of the macula, and visual cortical pathways were more fully captured by VEPs than visual acuity or DTI metrics.

**Supplementary Information:**

The online version contains supplementary material available at 10.1007/s10633-023-09927-w.

## Introduction

Abusive head trauma (AHT) including “shaken-baby syndrome” and “nonaccidental trauma” describes inflicted head injuries in young children [1]. AHT is among one of the common causes of death and disability among infants and children [2, 3]. The mechanism of cerebral injury in AHT can be due to impulsive-loading, impact-loading, or both [4]. Impulsive-loading refers to nonimpact forces generated by severe rotational angular velocity and acceleration/deceleration forces often associated with violent shaking [5]. Impulsive loading is thought to result in shearing injury to the brain and meninges. In contrast, impact-loading refers to direct application of blunt forces to the head that typically result in scalp injury, skull fracture and brain contusions with associated focal small extra-axial hemorrhages [4]. In AHT, findings at clinical presentation can include loss of consciousness, altered respiration, subsequent altered neurological function, extensive retinal hemorrhages, intracranial hemorrhage, subdural hematomas, cerebral edema, and ischemic changes on neuroimaging [6–8]. The reported history can be inconsistent with associated physical injuries. The type of retinal hemorrhages and their distribution help distinguish AHT from non-AHT causes of retinal hemorrhages [9]. Careful examination of the retina within 24–48 h of presentation is important to support the diagnosis of AHT because intra-retinal hemorrhages clear rapidly [9, 10].

Retinal hemorrhages from AHT are thought to result from multi-directional acceleration and deceleration forces causing traction between the retina and vitreous due to angular acceleration of the head on the neck [9, 11, 12]. Finite element analysis suggests that repetitive rotational cranial accelerations augment the forces at the vitreo-retinal interface at the regions where retinal hemorrhages occur in AHT [11]. Yet, retinal hemorrhages specificity for rotational cranial accelerations as in infant shaking remains unknown.

Histology at autopsy, and *in-vivo* optical coherence tomography (OCT), reveal a range of localized abnormalities such as vitreoretinal-interface detachment, perimacular folds, optic nerve sheath hemorrhage, retinal fibrotic scars, multilayered retinoschisis (or disruption across retinal layers), foveal detachment, and macular pseudo-holes [13–20]. During the acute phase, there are large variations in abnormal full-field and focal electroretinogram (ERG) responses [16, 21, 22]. A subset of subjects shows severely reduced ERG responses whereas about one-third have a normal ERG in one eye. The ERG abnormalities cannot be explained by retinal hemorrhages alone [22]. At follow-up examination, a majority of AHT victims have a normal fundus appearance while about 20% show evidence of “retinal scarring” or optic disc pallor [23]. Survivors of AHT who presented with extensive retinal hemorrhages and traumatic retinoschisis (TR) are thought to have a worse neurological outcome and lower survival rate [8, 21, 24, 25].

Diffusion weighted imaging and diffusion tensor imaging (DTI) are important in managing AHT cases. Diffusion weighted imaging is sensitive to restricted diffusion due to cerebral infarction, hypoxia–ischemia, cytotoxic edema, and possibly excitotoxic brain injury [4, 26, 27]. In comparison, DTI measures the directional water diffusion in brain tissue and is well suited to examine diffuse axonal injury [28–30] in infants with AHT. Specifically, rotational accelerating and decelerating forces can cause greater shearing of axons at the grey-white matter junction of an underdeveloped-myelinated brain. DTI relies on collinear arrangements of axons in white matter, which limits diffusion of water perpendicular to axon direction. A common DTI metric is fractional anisotropy (FA), which describes the directional coherence (anisotropy) of water diffusion within brain voxels. FA ranges from 0.0 (isotropic diffusion) to 1.0 (highly anisotropic along one direction). Other DTI metrics that describe the direction and magnitude of water diffusion in white matter tracts are mean diffusivity (MD), which measures overall magnitude of diffusion within tissue over three orthogonal directions. Axial diffusivity (AD) and radial diffusivity (RD) measure diffusion parallel and perpendicular to the principal axis of fibers, respectively. Traumatic brain injury in children shows both acute and long-term reductions in FA in multiple white matter tracts using DTI [28, 31, 32]. Microstructural alterations in AHT also include reductions in MD resulting from decreased AD, with preserved RD and FA [30]. Hypoxic/ischemic axonal injury may be another cause of altered diffusivity [33].

A poor visual outcome in AHT survivors is often attributed to cerebral visual impairment based on clinical neuroimaging [23, 34, 35]. However, assessment of the visual pathway using visual evoked potentials (VEPs) or visual acuities in AHT survivors are lacking. The primary purpose of this study is to evaluate visual function by behavioral assessment of visual acuity, visual pathway function using objective VEP analysis, and assess anatomy of white matter tracts within the occipital lobe and occipital gray matter using DTI metrics. Finally, we evaluated the relationship of retinal hemorrhages at presentation to subsequent outcomes of visual acuity, VEP, and DTI.

## Methods

Institutional Review Board approval from Seattle Children’s Hospital was obtained for retrospective review and thus informed consent was waived. Data analysis was performed in accordance with Institutional Review Board guidelines and regulations. The study adhered to the Declaration of Helsinki and procedures conformed to the US Health Insurance Portability and Accountability Act requirements. Determination of AHT, inclusion criteria, and exclusion criteria for AHT has been previously described [22]. AHT subjects had a full eye exam with dilated fundus examination within 1–2 days of presentation, cranial imaging by computed tomography and/or magnetic resonance imaging (MRI) in the acute phase, and follow-up vision assessment. Retinal imaging was supplemented in some subjects using a RetCam 3 (Clarity Medical Systems, Pleasanton, CA). Subjects with penetrating head injury or anterior segment injury of the eye were excluded.

Grouping of retinal hemorrhage patterns was based on retinal findings at presentation. Because retinal hemorrhage descriptions lacked standards across the retrospective dataset, we used simplified grouping criteria based on binocular indirect ophthalmoscopy and relevance to central vision. The first group comprised subjects presenting with a traumatic retinoschisis in either eye (*TR group*). Traumatic retinoschisis was defined as a splitting of retinal layers and/or the vitreoretinal surface associated with partial-thickness elevation of the retina with or without surrounding retinal folds. Cases with circumlinear retinal folds were included with traumatic retinoschisis. The second group included subjects lacking retinoschisis but who had retinal hemorrhage that at least involved the macula in either eye (Mac group) regardless of the number of hemorrhages in the periphery. The third group included subjects presenting with retinal hemorrhages solely outside of the macula in either eye (NoMac group). The fourth group included subjects presenting with no detectable retinal hemorrhages in either eye (NoHem group).

Visual function assessments at follow-up were analyzed if there was sufficient recovery time to clear retinal hemorrhages [36]. For those with traumatic retinoschisis and vitreous hemorrhages, data were acceptable if the assessment was at least 65 days after presentation (days of recovery). For those with any other type of retinal hemorrhages, data were acceptable if the assessment was at least 21 days of recovery. For the NoHem group there was no recovery time limit.

At follow-up, behavioral visual acuity in non-verbal children was tested by Teller Acuity Cards (Precision Vision, Woodstock IL) at 55 cm distance. In verbal children, visual acuity was tested by HOTV optotypes at 13 feet distance. For uniformity, all measurements were converted to log minimum angle of resolution (logMAR) and corrected for age if the subject was younger than 4 years [37]. The benefit of age-corrected logMAR is that a normal visual acuity value will be 0.0 regardless of visual development with age. Subjects without visual orienting to the low-vision card (approximately 20/2700 Snellen equivalent) were assigned a value of 3.0. Snellen ratio was not reported in this study because of potential differences between grating and recognition acuity. For reference, LogMAR values can be converted to age-corrected Snellen equivalent using Snellen ratio = 20/ 20*10^logMAR^. All logMAR data analyses were evaluated by nonparametric statistics (Mann–Whitney U test and Spearman rank correlation).

VEP data were included if testing was done after the criterion recovery period and within a year of presentation. Procedures for VEP recording and analysis have been published [38, 39]. In brief, VEPs were recorded under binocular viewing. Recording sites were standard 10–20 locations (Oz, O1, O2, T5, T6, Inion) referenced to Cz with ground at Pz. Stimuli were reversing checkerboards of large size and high contrast to accommodate severe vision impairment (163 arc minutes, 80% contrast, 2.74 reversals per second).. To counter the abnormal fixation or seizures in these subjects, objective analytical methods were used [37–39]. The electroencephalogram was carefully reviewed to remove spikes or large background noise by visual inspection then followed by standard averaging. All individual stimulus trials then underwent discrete Fourier transforms to derive an objective signal-to-noise ratio (SNR) based on statistics of the phase-amplitude distribution of multiple harmonics. A large SNR indicates underlying VEP responses have large amplitude components with accurate time-locking to the stimulus presentation. A SNR ≤ 1.3, was not statistically different from background noise. Averaged VEPs were digitally filtered 1.5–41.3 Hz, then scored for latency (time to the prominent positive peak after 80 ms) and amplitude (voltage difference between this peak and the preceding negative deflection if present, or baseline). Control data were taken from published data using an identical analysis [38].

All MRIs were reviewed by a pediatric neuroradiologist masked to the visual outcomes. Details of MRI and DTI protocols have been previously published[39]. MRI sequences included T1 magnetization-prepared rapid acquisition of gradient echo, T1- and T2-weighted sequences, and axial fluid‐attenuated inversion recovery (FLAIR). DTI data and deterministic fiber-tractography were analyzed using DSI Studio (http://dsi-studio.labsolver.org) [40]. Tracking parameters were default anisotropy threshold (qa), 60° angular threshold, 0.1 mm step size, track length between 30–400 mm, and a maximum of 10,000 seeds. DSI Studio performed automatic seeding of white matter tracts based on the ICBM152 tractography atlas with an internal fractional anisotropy threshold. If automated tracking failed, the fa map was manually re-seeded. Fiber tracking was chosen for relevant white matter tracts in the occipital lobe: optic radiations, inferior fronto-occipital fasciculus, and vertical occipital fasciculus. If automated tractography failed to find a specific tract, manual seeding was performed by drawing regions of interest on the FA images. All tracts were reviewed and fibers and clearly aberrant pathways or overlapping tracts were removed. We report average fractional anisotropy (FA) and mean diffusivity (MD) of reconstructed tracts only as post-hoc analysis revealed high intercorrelations between MD, RD, and AD. A secondary analysis measured MD using region of interest volumes of striate/extra-striate cortex (V1 and V2) by automated registration and warping. DTI metrics from each hemisphere was analyzed with respect to the VEP from the ipsilateral electrode. For example, results from the O1 electrode were paired with DTI metrics from the left hemisphere, and the O2 electrode was paired with the right hemisphere. Due to clinical restraints of the MRI, there was no criteria for timing of the DTI scan after presentation. DTI metrics were compared to VEPs if both tests were performed within age ranges that VEPs are stable [38, 41], which is within 1 month for subjects 4–6 months old, or within 1 year for subjects > 6 months. Controls for DTI imaging used the same MRI scanners, matched for age, and analyzed under identical parameters [39]. Of note, VEPs were not collected on DTI controls.

Statistical analysis was performed by Excel 365 (Microsoft, Redmond WA) or SPSS version 12 (Armonk, NY). We used the t-test (two-tail) for samples of unequal variance. Multiple group comparisons with unequal variance and unequal sample sizes were compared using Tamhane’s T2 test. *P*-values ≥ 0.02 were discarded as statistically non-significant owing to the small sample sizes and multiple post-hoc comparisons.

## Results

Forty-five of 202 children who presented with AHT between December 1996 and December 2017, met inclusion criteria. Age at presentation ranged from 24 days to 4.9 years (average 226 days; median 131; interquartile range 65–215). Thirty-seven of the subjects received initial care for AHT at our institutions, physical abuse data from the remaining eight subjects were gleaned from dictated records. Six subjects (13%) fell into the NoHem group. All in the NoHem group presented with acute neurological symptoms and no evidence of chronic SDHs. Seventeen subjects (38%) fell into the NoMac group, nine subjects (20%) fell into the Mac group, and twelve subjects (27%) fell into the TR group. One subject in the NoMac group subsequently died from complications of their CNS injury.

Table 1 shows the incidence of physical and clinical findings for each retinal grouping. Scalp Injury includes visually apparent scalp or brow bruising and scalp swelling due to edema, subgaleal hemorrhage or cephalhematoma. Impact injury includes scalp injury or skull fracture. Non-cranial injury includes bruising, burns, subconjunctival hemorrhage, oral injuries abdominal trauma or non-skull fractures. Of note, none of the subjects in the TR group had evidence of a skull fracture. Also, there was a high incidence of skull fracture (43%) and other impact injuries in the NoHem group. Neurological or cognitive deficits (seizures, abnormal developmental delay, attention deficit hyperactive disorder) were present in each group. Note that all subjects in the TR group had a cognitive deficit (abnormal developmental delay/attention deficit hyperactivity disorder) and were more likely to have a motor deficit (cerebral palsy, spasticity, dystonia, or partial paresis).

**Table 1:** Results based on retinal hemorrhage pattern at presentation

	TR	Mac	NoMac	NoHem
Age at presentation (years; mean (SD))	0.4 (0.2)	0.5 (0.5)	0.9 (1.3)	0.4 (0.6)
Age at last F/U (years; mean (SD))	4.8 (3.1)	2.2 (2.3)	1.9 (3.6)	3.5 (2.9)
N	12	9	17	7
SDH	92%	89%	100%	57%
Skull fracture	0%	33%	6%	43%
Scalp injury	8%	33%	18%	57%
Impact injury	8%	33%	24%	57%
Non-cranial injury	75%	67%	53%	57%
Seizures/Ep	42%	67%	24%	57%
DD / ADHD	100%	67%	59%	86%
Motor deficit	58%	22%	41%	43%

*IQR interquartile range, SDH* subdural hemorrhage, *DD/ADHD *developmental delay or attention deficit hyperactivity disorder, retina grouping, *TR *traumatic retinoschisis, *Mac *retinal hemorrhage involving the macula, *NoMac *retinal hemorrhages outside of the macula, *NoHem *no retinal hemorrhages

**Fig. 1:**
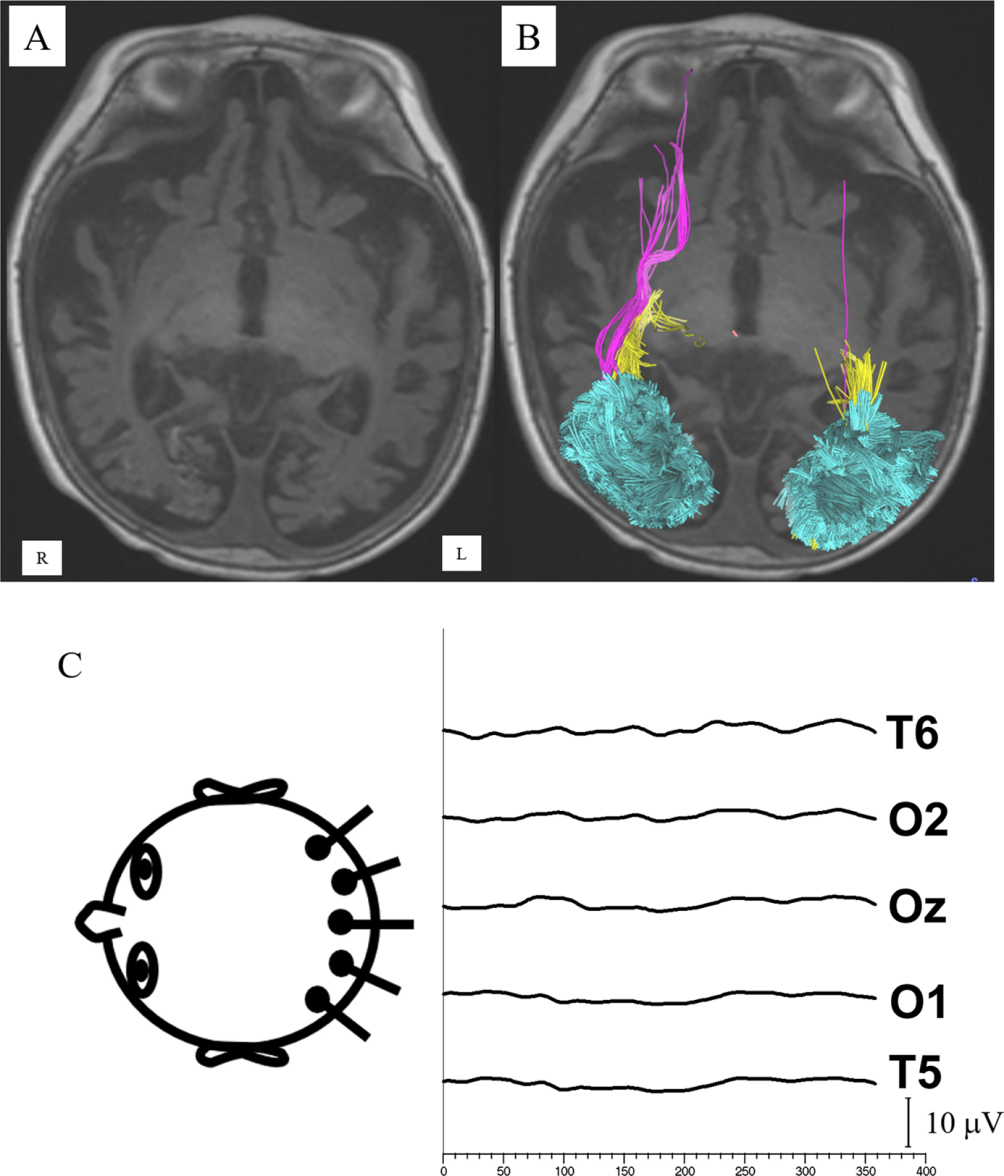
A child presenting at 108 days age with unresponsiveness, respiratory distress, subarachnoid hemorrhage, rib fractures and abdominal injury, but normal appearing fundus in both eyes. **A** VEP at 0.5 years age showed responses near background noise levels. MRI at 1 year of age showed significant cerebral volume loss, laminar necrosis within the frontal, parietal, and occipital lobes. Diffusion tensor imaging showed reduced volume of the optic radiations (yellow), reduced volume of the inferior fronto-occipital fasciculus (purple), and mild volume loss of the vertical occipital fasciculus. (cyan). At last follow-up, visual acuity was 1.53 logMAR and he had global developmental delay, intractable epilepsy, and spastic quadriparesis. **A** Axial T1-weighted image, **B** same image with reconstructed tracts superimposed, **C** VEP responses across the occiput

**Fig. 2:**
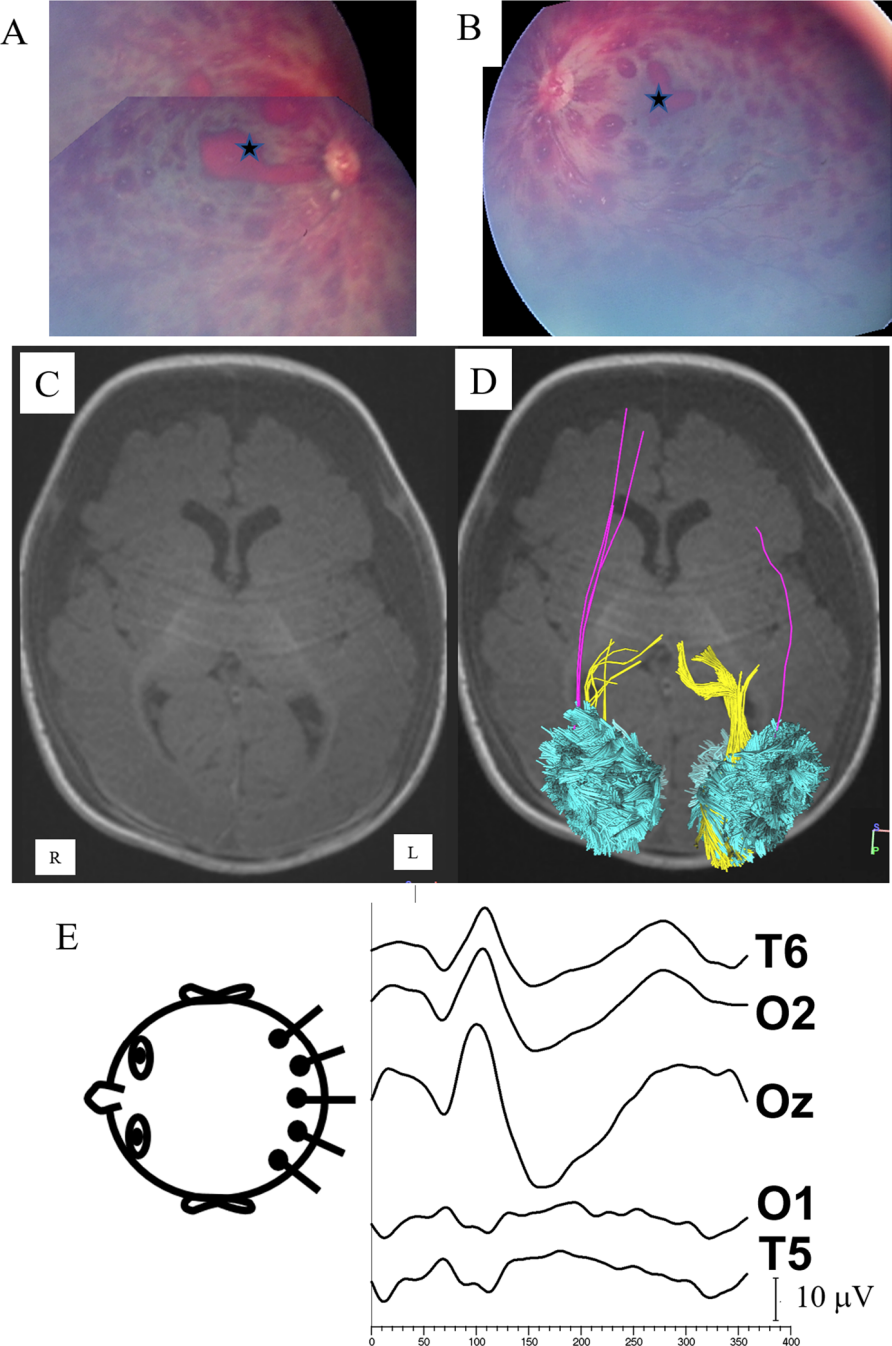
A child presenting at 151 days age with acute respiratory failure, status epilepticus, acute on chronic subdural hematomas, skull fracture, and leg fractures. RetCam imaging of the right eye **A** and left eye **B** show numerous pre- and intra-retinal hemorrhages with white centers, hemorrhage within both macula (stars), diffuse macular edema with sub-hyaloid hemorrhages. An MRI at 152 days age showed diffusion restriction on neuroimaging with areas of edema. **C** Axial T1-weighted image, **D** same image with reconstructed tracts superimposed showing reduced volume of the right optic radiations (yellow), bilateral reduced volume of the inferior fronto-occipital fasciculus (purple), and mild volume loss of the vertical occipital fasciculus. (cyan). E, VEP at 0.5 years age showed a larger response over the right hemisphere (paradoxical lateralization). At last follow-up visual acuity was 0.07 logMAR and he had seizures, mild developmental delay, and spastic left hemiparesis

**Fig. 3:**
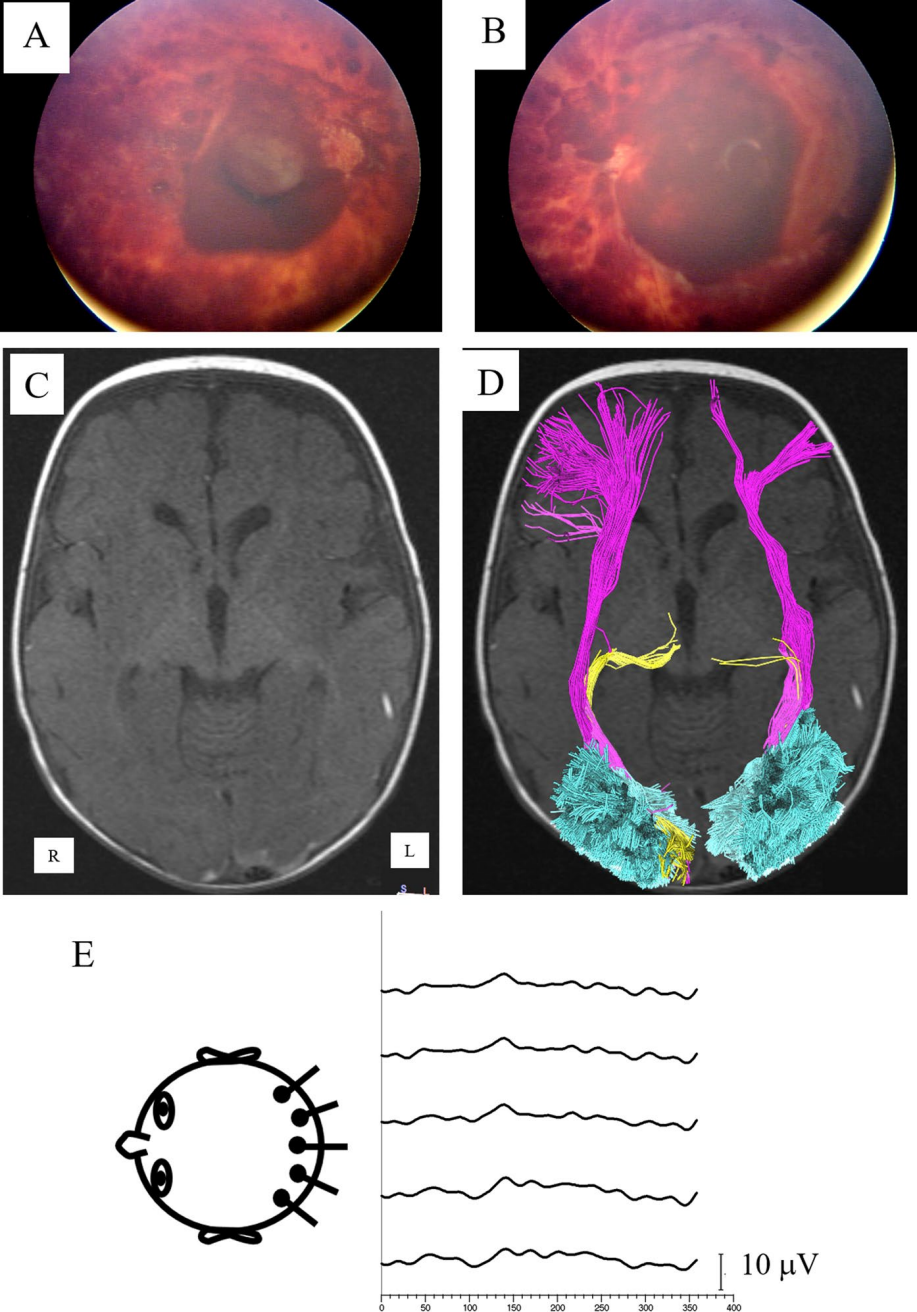
A child presenting at 133 days age with unresponsiveness, seizure-like episodes, subdural hematomas, skull fracture, and leg fractures. RetCam imaging of the right eye **A** and left eye **B** show numerous intraretinal, preretinal, subretinal hemorrhages. Both eyes had a traumatic schisis cavity with associated circumferential folds. Both optic discs were swollen. An MRI at 136 days age showed diffuse abnormal FLAIR signal, restricted diffusion within both cerebral and cerebellar hemispheres. **C** Axial T1-weighted image, **D** same image with reconstructed tracts superimposed showing reduced volume of the left optic radiations (yellow), intact inferior fronto-occipital fasciculi (purple), and intact volume loss of the vertical occipital fasciculi. (cyan). E, VEP at 0.7 years age showed significantly reduced responses of delayed latency. At last follow-up visual acuity was 0.86 logMAR and he had global developmental delay and autism disorder

At last dilated examination, twenty-seven subjects (60%) had a normal fundus. Of the remaining eighteen subjects with an abnormal fundus, fifteen subjects had evidence of optic atrophy (including mild temporal pallor), seven subjects had both optic atrophy and macular abnormalities, and three subjects had abnormalities of the macula only (i.e., scarring, pigmentary mottling). The percentage of abnormal fundus findings for each group is shown in Table 2. Representative data from subjects in the NoHem, Mac, and TR groups are shown in Figs. 1, 2 and 3.

**Table 2:** Functional and anatomical results

	TR	Mac	NoMac	NoHem	Controls
Optic atrophy	67%	44%	14%	14%	–
Abnormal macula	42%	44%	10%	0%	–
*Mean (s.d.) visual acuity at last follow-up, in age-corrected logMAR*^*a*^					
Age (years)	5.2 (3.2)	2.9 (2.5)	3.3 (4.3)	4.0 (2.5)	–
Acuity	1.8 (1.1)	0.6 (1.0)*	0.9 (1.1)	1.7 (1.3)	–
No F&F	42%	11%	18%	43%	–
*Mean (s.d.) visual evoked potentials*					
Age, years	0.9 (0.5)	1.0 (0.6)	1.3 (1.4)	0.9 (0.5)	1.1 (1.0)
N	8	7	17	6	24
Amplitude	2.4 (2.5)***	5.3 (4.2)***	13.2 (12.2)	6.8 (6.9)*	24.7 (10.4)
Latency	135 (33)	112 (19)	119 (47)	104 (14)	104 (7)
SNR	3.7 (7.3)***	28.3 (25.9)*	42.9 (42.2)	31.4 (29.0)	84.9 (48.1)
No signal	75%	14%	18%	33%	–
*Means (s.d.) diffusion tensor imaging*^*b*^					
Age	0.5 (0.1)	0.3 (0.1)	2.7 (2.7)	0.9 (0.6)	1.1 (1.1)
N	6	5	4	4	33
FA	0.236 (0.011)	0.190 (0.012)**	0.225 (0.013)	0.236 (0.013)	0.229 (0.005)
MD	1.00 (0.03)	1.10 (0.03)	1.17 (0.04)	1.19 (0.04)	1.06 (0.02)
Volume (mm^3^)	10,355 (2045) *	10,104 (2240)*	15,062 (2504)	13,178 (2504)	17,181 (1024)

Retina grouping: *TR *traumatic retinoschisis, *Mac *retinal hemorrhage involving the macula, *NoMac *retinal hemorrhages outside of the macula, *NoHem *no retinal hemorrhages

^a^*LogMAR *logarithm of the minimum angle of resolution, comparisons using Mann–Whitney U test versus No Hem group. *No F&F *no visual tracking or orienting to 20/2700 target

^b^All tracts combined.* FA *fractional anisotropy, *MD *mean diffusivity

Comparisons using Tamhane’s T2 assuming unequal variances; *p*-value vs controls

*** < 0.0001, ** < 0.001, * < 0.01

### Visual acuity

**Fig. 4:**
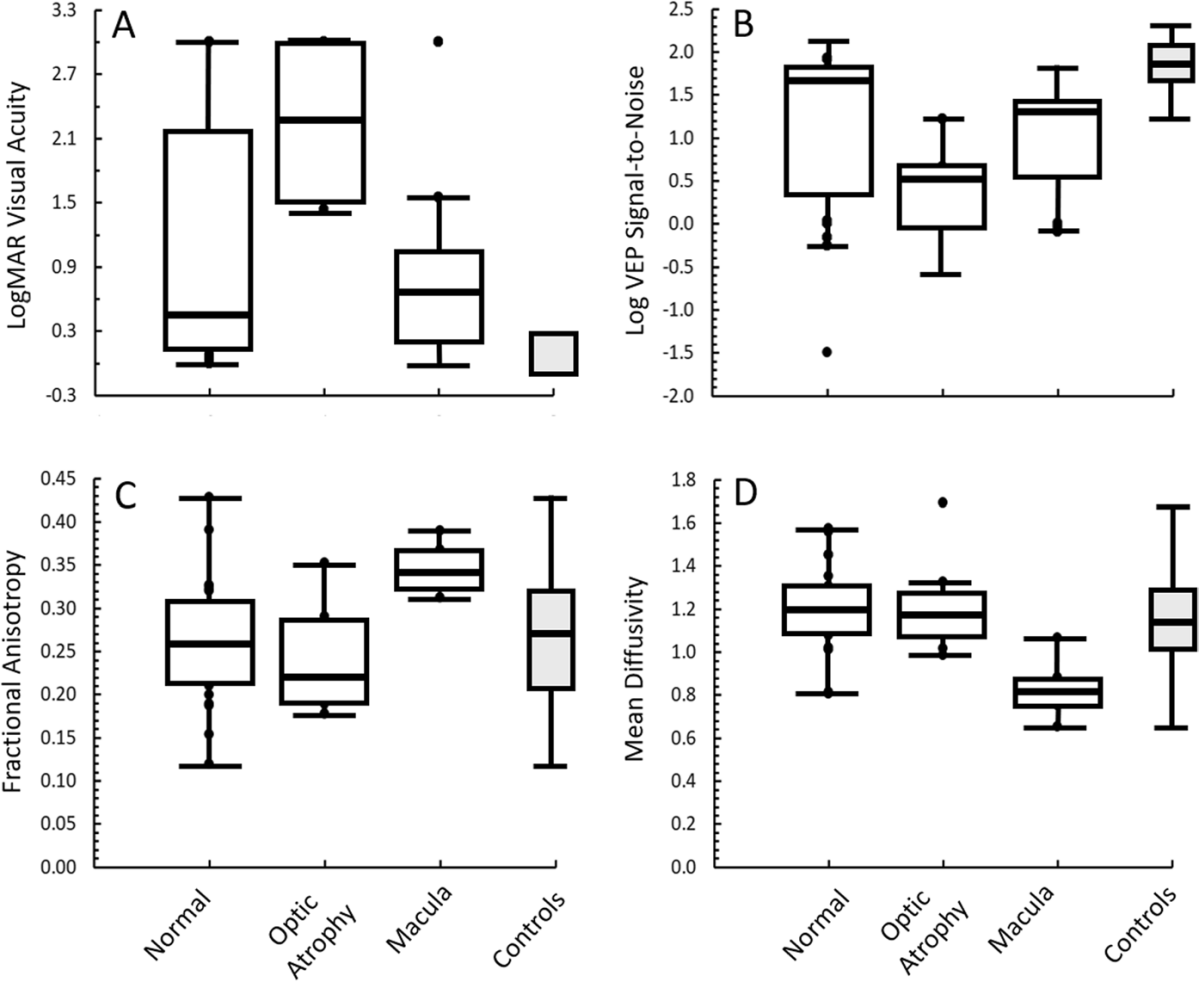
Boxplots of results based on dilated fundus examination at last follow-up. Subjects were grouped based on a normal examination, presence of optic atrophy, or presence of abnormal pigmentary mottling/scarring within the macula. Age-appropriate controls are shown at right in each plot in the shaded box. **A** visual acuity represented by logarithm of minimum angle of resolution (logMAR) after correcting for age. The range for controls is the clinically accepted range of normal. **B** Logarithm of visual evoked potential (VEP) signal-to-noise ratios. **C** Fractional anisotropy from diffusion tensor imaging of the optic radiations. **D** Mean diffusivity from diffusion tensor imaging of the optic radiations

At last follow-up, average age was 3.8 years (median 3.2; quartile range 1.1–5.3). Thirteen subjects (29%) had an age-corrected logMAR visual acuity within normative range (logMAR < 0.3). Twelve subjects (27%) had severe vision loss with no visual orienting to the low-vision card (logMAR = 3.0). Median logMAR was 0.8 (first quartile = 0.20, third quartile = 3.0). Results for each group are shown in Table 2. The TR and NoHem groups had the worst outcomes on visual acuity at last follow-up. Based on the fundus examination at the last follow-up, subjects with optic atrophy had statistical worse vision than subjects with a normal examination (*p* = 0.008). There was no difference (*p* = 0.80) between those with a normal examination versus those with macular abnormalities (Fig. 4).

### Visual evoked potentials

VEPs meeting inclusion criteria were available in 38 of the 45 subjects. Age at VEP testing ranged from 146 days—5.2 years (average 1.1 years; median 0.64 year; interquartile range = 0.5–1.2). VEP amplitude at the Oz electrode was highly correlated with SNR (*r* = 0.91; *p* < 0.0001) indicating the objective SNR analysis captured the variance of the subjective scoring of amplitude. Twenty-four controls from a prior study [38] were selected to match the age range of the subjects (t-test group ages; *p* = 0.99). Compared to controls, AHT subjects had reduced amplitude (8.5 vs 24.7 µV, *p* < 0.0001), reduced SNR (30.1 vs 84.9, *p* < 0.0001) and minimal latency prolongation (118.9 vs 103.9 ms, n.s.). Twelve subjects (32%) had a SNR below background noise (≤ 1.3) indicating no detectable signal. Overall, twenty of the AHT subjects (53%) had a SNR below that measured in any control.

VEP results for each group are shown in Table 2. The TR group had the poorest VEP outcomes, followed by the Mac group. The NoMac and NoHem groups were not statistically different from controls on almost all VEP metrics. Thirty-three AHT subjects had both VEPs and follow-up visual acuity measurements. Age-corrected logMAR at last follow-up was correlated with VEP amplitude (Spearman rank, *r* = − 0.683; *p* = 0.00001) and VEP SNR *r* = − 0.690; *p* = 0.00001).

Based on the fundus examination at the last follow-up, subjects with optic atrophy had reduced VEP amplitude and SNR compared to subjects with a normal examination (*p* < 0.001 for both). There was no difference (*p* > 0.10) between those with a normal examination versus those with macular abnormalities (Fig. 4). Results for VEP amplitude were similar to that for SNR. There were no differences in VEP latency between subjects with a normal fundus examination versus those with optic atrophy or abnormalities of the macula (*p* > 0.69).

### Diffusion tensor imaging and tractography

**Fig. 5:**
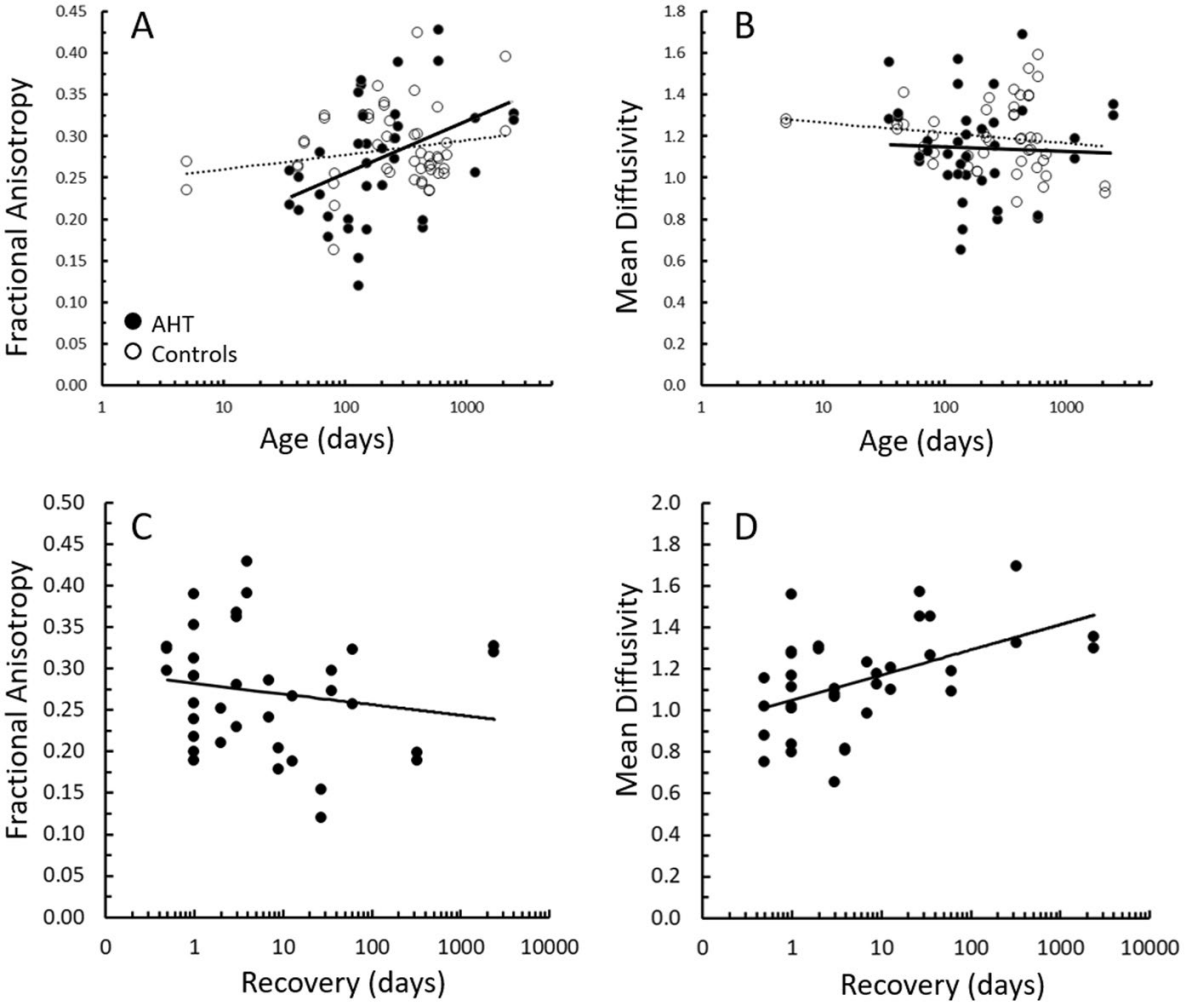
Diffusion tensor imaging results. Relationship between fractional anisotropy **A** and mean diffusivity **B** versus log age for the optic radiations. Filled circles and solid regression lines are for subjects with history of abusive head trauma. Open circles and dotted regression lines are for controls of similar age range. Relationship between fractional anisotropy **C** and mean diffusivity **D** versus recovery duration for the optic radiations for subjects with history of abusive head trauma

For all white matter tracts combined, FA was reduced in the Mac group, but otherwise there was no significant effect of retinal hemorrhage pattern at presentation with FA or MD (Table 2). Both the TR and Mac groups had lower white matter volumes than controls. Previous work has reported DTI metrics (FA, MD, AD, and RD) change with log age [39, 42, 43]. Our controls also showed significant changes in DTI metrics with log age for the vertical occipital fasciculus and inferior fronto-occipital fasciculus, and area V2 (Online Resource 1). In comparison, AHT subjects had weak relationships of DTI metrics with log age. Summary DTI data are shown in Fig. 4 for the optic radiations. AHT subjects showed evidence that MD was related with the duration of recovery at the time of the DTI in the optic radiations (Fig. 5 and Online Resource 1).

Overall, DTI metrics were not statistically correlated with logMAR or VEP metrics (Online Resource 1). Further inspection of the data showed that subjects with severely reduced VEPs (SNR < 1.4) had a wide range of MD values. Additionally, there were no significant relationships if VEP and DTI metrics were compared with respect to left/right hemisphere ratios (correlations ranged from − 0.357–0.085; *p* > 0.05 for all comparisons). RD, which is an index of white matter integrity, was not related to VEP latency (*r* = − 0.18). Also, left/right hemisphere ratios of RD were not related to left/right hemisphere differences of VEP latency (*r* = − 0.06). There was no relationship between age at the time of VEP and the age at the time of DTI, which was related to neuroimaging performed during the acute phase. Based on the fundus examination at the last follow-up, there were no differences in tract volume or MD between subjects with a normal fundus examination versus those with optic atrophy or macular abnormalities. FA was statistically higher, and MD was statistically lower, in subjects with macular abnormalities (*p* < 0.01) compared to those with a normal fundus examination or those with optic atrophy (*p* < 0.004; Fig. 4).

Because age and recovery showed effects on DTI metrics, a post-hoc analysis of covariance (ANCOVA) was performed using age and duration of recovery as covariables. All tracts were compared between AHT subjects and controls. AHT subjects had an increase in MD (1.12 in AHT vs 1.0 in controls; *p* < 0.001) and a decrease in AD (1.34 in AHT vs 1.40 in controls; *p* = 0.013). Also, there was a significant decrease in DTI tract volumes in AHT subjects (12,017 mm^3^ in AHT vs 17,167 mm^3^ in controls; *p* < 0.001). Finally, a multiple regression examined recovery times across dependent variables. The regression included 1) days of recovery at DTI acquisition, 2) days of recovery at VEP recording, 3) FA after adjusting for age, 4) MD after adjusting for age, 5) DTI volume adjusting for age, and 6) retinal hemorrhages in the macula at presentation (TR and Mac versus NoMac and NoHem groups as they require longer recovery). The regression model for VEP SNR resulted in a non-significant prediction (*R*^2^ = 0.194) and the regression model for age-corrected logMAR resulted in a non-significant prediction (*R*^2^ = 0.06).

## Discussion

This study provided detailed evaluations of the visual system in children with AHT. We found a high incidence of vision impairment in children with AHT, consistent with prior reports using subjective clinical assessments [23, 25]. In contrast, this study utilized objective VEP data, age-corrected visual acuity, and assessment of diffusion metrics in the occipital lobe. Visual acuity was significantly reduced in 71% of AHT victims with 27% having no visual orienting. By VEP testing, 53% had a severely reduced VEP response with 32% having no detectable signal. Our main finding was that VEPs indicated a worse visual outcome in subjects initially presenting with traumatic retinoschisis or hemorrhages involving the macula. The VEP data, but not visual acuity, were consistent with the notion that a traumatic retinoschisis is associated with a poorer visual outcome [8, 21, 25, 34]. The discrepancy between visual acuity and VEP findings is likely related to differences in what each test measures. For instance, visual acuity assessment in children is dependent upon multiple factors such as attention, cognitive status, visual orienting in non-verbal subjects, integrity of the retina, and relative preservation of peripheral visual field. In contrast, the VEP is an objective response that primarily reflects a population response from the central 20 degrees of the visual field in striate and extra-striate cortex [44–46].

Vision impairment in non-verbal children with AHT is often attributed to cerebral visual impairment when the child shows poor visual orienting behaviors in the context of a normal fundus examination and neurological impairment [23, 34]. This study used VEPs and DTI to objectively assess for cerebral visual impairment in the occipital lobe. If cerebral visual impairment was the primary cause of vision loss, then the VEP and DTI metrics were expected to show an association. However, diffusion metrics on DTI tractography were not associated with VEP measures and were not associated with visual acuity outcomes. Possible reasons for the discrepancy are 1) changes in brain diffusion during the recovery period, 2) technical challenges in acquiring DTI signals in young unmyelinated brains, and 3) variations in clinical parameters of DTI acquisition. Given the nature of clinical neuroimaging, the timing of the DTI imaging after presentation varied greatly and could not be controlled. Another issue is whether the predominant parenchymal injury in children with AHT is hypoxic-ischemic injury rather than traumatic diffuse axonal injury [4]. An important caveat with DTI is that FA, AD, MD reflect variable biological changes including edema, inflammation, or superimposed crossing fibers within a voxel. These mechanisms could be independent of the electrophysiological population response from visual cortex.

Furthermore, if cerebral visual impairment is the primary mechanism of vision loss in AHT subjects, then DTI metrics are expected to be significantly abnormal in subjects who have a normal retinal examination. We found DTI metrics were within the range of controls (albeit at the extreme ends of control values) for subjects presenting with a normal appearing retina, and for those with a normal appearing retina on follow-up. In contrast, both VEP and DTI metrics were more affected in subjects who presented with either a traumatic retinoschisis, or hemorrhage involving the macula, or who subsequently were found to have macular abnormalities on follow-up. The common factor across these findings is damage to the macula within the retina. Specifically, traumatic retinoschisis or macular hemorrhages often cause abnormalities in retinal architecture of the fovea and macula on OCT [13–18]. Future work using the OCT can help to elucidate the role of retinal anatomy to vision loss in children with AHT.

The frequency of a traumatic retinoschisis in one or both eyes in this study was 25%, which is similar to that reported in prior studies [8, 24, 34]. Of interest, none of the subjects with a traumatic retinoschisis had evidence of skull fractures, which is consistent with the notion that retinoschisis is associated with repeated cranial angular acceleration as opposed to impact trauma. This suggests a relationship between rotational angular acceleration of the head on the neck, as in infant shaking, with vitreo-retinal traction. Case studies using the OCT [13–15, 18] support the findings of hemorrhagic detachment of the internal limiting membrane and vitreoretinal traction underlying perimacular folds in abusive head trauma [19, 20]. Further studies will be needed to determine the association of acceleration–deceleration forces versus evidence of impact trauma with the findings of the vitreoretinal interface. The data in our study suggest the mechanisms causing traumatic retinoschisis, or trauma causing long-term abnormalities of the macula, result in greater visual system dysfunction.

There are several potential limitations of this study. First the data is retrospective from an observational dataset that also used documentation that includes providers outside our institution. Not all subjects had follow-up studies due to the complexity and social issues associated with AHT. Additionally, it can be difficult detecting retinoschisis beneath concurrent pre-retinal or vitreous hemorrhages.

## Summary statement

This study shows that the VEP in pediatric abusive head trauma leads to more information about visual outcome than visual acuity or diffusion tensor imaging on MRI. Different patterns of retina hemorrhages at presentation likely reflect different types of injuries with different visual outcomes. For instance, traumatic retinoschisis, likely caused damage by angular acceleration/deceleration, often resulted in severe visual loss even though none of the patients had obvious external head trauma. A group of subjects that showed no obvious retinal trauma, but obvious impact trauma (fractured skull, scalp) also resulted in severe visual loss. Furthermore, the study suggests visual loss attributed to cortical injury could occur from damage earlier in the visual system.

## Supplementary Information

Below is the link to the electronic supplementary material.

Supplementary file1 (DOCX 18 KB)

## Abbreviations

AD: Axial diffusivity (diffusion parallel to axis of axons on diffusion tensor imaging)
AHT: Abusive head trama
DTI: Diffusion tensor imaging (an advanced magnetic resonance imaging technique)
ERG: Electroretinography
FA: Fractional anisotropy (directional diffusion tensor imaging)
FLAIR: Axial fluid‐attenuated inversion recovery (magnetic resonance imaging sequence)
logMAR: Logarithm of minimum angle of resolution (visual acuity)
MAC: Macula of the retina
MRI: Magnetic resonance imaging
OCT: Optical coherence tomography
RD: Radial diffusivity, (diffusion perpendicular to axis of fibers on diffusion tensor imaging)
SNR: Signal-to-noise ratio of the visual evoked potential
TR: Traumatic retinoschisis of the retina
VEP: Visual evoked potentials
V1: Striate cortex
V2: Extra-striate cortex
