# Supplementary material for: Pediatric abusive head trauma: visual outcomes, evoked potentials, diffusion tensor imaging, and relationships to retinal hemorrhages
Source: Doc Ophthalmol. 2023 Mar 7;147(1):1–14. doi: 10.1007/s10633-023-09927-w (PMC10329619; doi:10.1007/s10633-023-09927-w)
Supplement: Supplementary file 1 — Supplementary file1 (DOCX 18 KB) [file 10633_2023_9927_MOESM1_ESM.docx]

| Online Resource 1. Correlations for Diffusion Tensor Imaging | | | | | | | |
| --- | --- | --- | --- | --- | --- | --- | --- |
|  | CONTROLS | AHT | AHT | AHT | AHT | AHT | AHT |
|  | Log Age | Log Age | Recovery Days | logMAR | VEP Amplitude | VEP Latency | VEP SNR |
| Optic Radiations | | | | | | | |
| FA | 0.2028 | 0.401 | -0.175 | 0.240 | 0.100 | 0.177 | -0.043 |
| MD | -0.1640 | -0.049 | 0.503* | -0.275 | 0.084 | -0.162 | 0.146 |
| AD | -0.1519 | 0.08 | 0.528 ** | -0.301 | 0.163 | -0.135 | 0.146 |
| RD | -0.1649 | -0.119 | 0.470* | -0.318 | 0.035 | -0.170 | 0.112 |
| Vertical Occipital Fasciculus (VOF) | | | | | | | |
| FA | 0.6229 ** | 0.417* | 0.179 | 0.239 | 0.249 | 0.253 | 0.136 |
| MD | -0.9092 ** | -0.076 | 0.200 | -0.408 | 0.023 | -0.162 | 0.085 |
| AD | -0.8655 ** | -0.018 | 0.259 | -0.419 * | 0.093 | -0.124 | 0.146 |
| RD | -0.9114 ** | -0.107 | 0.166 | -0.395 | -0.017 | -0.183 | 0.049 |
| Inferior Fronto-Occipital Fasciculus (IFOF) | | | | | | | |
| FA | 0.7381 ** | 0.534 ** | 0.004 | 0.121 | 0.354 | 0.152 | 0.208 |
| MD | -0.8648 ** | -0.347 | 0.168 | -0.393 | -0.066 | -0.246 | 0.095 |
| AD | -0.8115 ** | -0.201 | 0.196 | -0.371 | 0.119 | -0.217 | 0.243 |
| RD | -0.8627 ** | -0.405 | 0.143 | -0.334 | -0.159 | -0.245 | 0.011 |
| Visual Cortex V1 Region of Interest | | | | | | | |
| MD | -0.295 | 0.172 | 0.639 ** | -0.04 | -0.183 | 0.04 | -0.238 |
| Visual Cortex V2 Region of Interest | | | | | | | |
| MD | -0.474 ** | 0.099 | 0.602 ** | -0.096 | -0.170 | -0.007 | -0.197 |
| AHT= subjects with abusive head trauma; FA = fractional anisotropy; MD= mean diffusivity; AD = axial diffusivity; RD = radial diffusivity; SNR = signal-to-noise ratio; Recovery Days = duration from presentation to the time of brain imaging; ** p< 0.001; * p< 0.01 | | | | | | | |
